# High-Throughput Sequencing and Copy Number Variation Detection Using Formalin Fixed Embedded Tissue in Metastatic Gastric Cancer

**DOI:** 10.1371/journal.pone.0111693

**Published:** 2014-11-05

**Authors:** Seokhwi Kim, Jeeyun Lee, Min Eui Hong, In-Gu Do, So Young Kang, Sang Yun Ha, Seung Tae Kim, Se Hoon Park, Won Ki Kang, Min-Gew Choi, Jun Ho Lee, Tae Sung Sohn, Jae Moon Bae, Sung Kim, Duk-Hwan Kim, Kyoung-Mee Kim

**Affiliations:** 1 Department of Pathology, Samsung Medical Center, Sungkyunkwan University School of Medicine, Seoul, Korea; 2 Department of Medicine, Division of Hematology-Oncology, Samsung Medical Center, Sungkyunkwan University School of Medicine, Seoul, Korea; 3 Department of Surgery, Samsung Medical Center, Sungkyunkwan University School of Medicine, Seoul, Korea; 4 Cancer Companion Diagnostics Center, Samsung Medical Center, Seoul, Korea; 5 Department of Molecular Cell Biology, Samsung Biomedical Research Institute, Sungkyunkwan University School of Medicine, Suwon, Korea; Sapporo Medical University, Japan

## Abstract

In the era of targeted therapy, mutation profiling of cancer is a crucial aspect of making therapeutic decisions. To characterize cancer at a molecular level, the use of formalin-fixed paraffin-embedded tissue is important. We tested the Ion AmpliSeq Cancer Hotspot Panel v2 and nCounter Copy Number Variation Assay in 89 formalin-fixed paraffin-embedded gastric cancer samples to determine whether they are applicable in archival clinical samples for personalized targeted therapies. We validated the results with Sanger sequencing, real-time quantitative PCR, fluorescence in situ hybridization and immunohistochemistry. Frequently detected somatic mutations included *TP53* (28.17%), *APC* (10.1%), *PIK3CA* (5.6%), *KRAS* (4.5%), *SMO* (3.4%), *STK11* (3.4%), *CDKN2A* (3.4%) and *SMAD4* (3.4%). Amplifications of *HER2*, *CCNE1*, *MYC*, *KRAS* and *EGFR* genes were observed in 8 (8.9%), 4 (4.5%), 2 (2.2%), 1 (1.1%) and 1 (1.1%) cases, respectively. In the cases with amplification, fluorescence in situ hybridization for *HER2* verified gene amplification and immunohistochemistry for HER2, EGFR and CCNE1 verified the overexpression of proteins in tumor cells. In conclusion, we successfully performed semiconductor-based sequencing and nCounter copy number variation analyses in formalin-fixed paraffin-embedded gastric cancer samples. High-throughput screening in archival clinical samples enables faster, more accurate and cost-effective detection of hotspot mutations or amplification in genes.

## Introduction

While gastric cancer is the fourth most common cancer in the world, it is the second leading cause of death. [1] Its incidence is significantly higher in Asian countries, including Korea, where it is the second most common cancer. [2] Recently, several targeted therapeutics for gastric cancer have been discovered, which provide additional options for physicians and patients [3]–[5].

In the era of targeted therapy, mutation profiling of the causative cancer is crucial for therapeutic decisions. Attempts to profile mutations have been made using traditional Sanger sequencing; however, it is not an optimal method in clinical settings due to the cost, time and labor required. Moreover, Sanger sequencing requires substantial amounts of DNA; evaluating small amounts of specimen for several genes at the same time is not possible. Introduction of next generation sequencing (NGS) methods has resolved this problem by multiplex, high-throughput sequencing of many samples for multiple genes simultaneously. [6], [7] One of the NGS platforms, the Ion Torrent AmpliSeq Cancer Panel, relies on non-optical detection of hydrogen ions in a semiconductor device, [8] and is able to detect 2,855 oncogenic mutations in 50 commonly mutated genes (Table S1). It is superior to other mass spectroscopy-based sequencing methods, providing sequencing results faster and at lower cost. [8] It is applicable in formalin-fixed paraffin-embedded (FFPE) tissue specimens with small amounts of DNA. Because it ensures high sensitivity in screening known oncogenic mutations, [9], [10] the Ion Torrent AmpliSeq Cancer Panel is the choice of 5 major cancer centers in the United States for molecular diagnostics in targeted therapy [11].

Amplification of oncogenes is a major mechanism for gene overexpression and contributes to tumor development. [12] Examples include amplification of *HER2*, *MET*, *FGFR2* and *KRAS* genes in gastric cancers. [13], [14] In the detection of copy number variations (CNVs) in clinical samples, fluorescence in situ hybridization (FISH) and/or immunohistochemistry (IHC) has been widely used. However, high costs and small sample sizes of biopsy materials limit the application of these methods, and there is still a need for further high-throughput technology with easy accessibility, high sensitivity and low costs. nCounter CNV CodeSets (Nanostring technologies, Life Sciences, Seattle, WA) provide superior accuracy and reproducibility for studies of all sizes and produce better, faster results with substantially less effort than with real-time quantitative polymerase chain reaction (qPCR) or CNV arrays [15].

Better-tailored cancer treatment may improve patient outcome. Patient tumor samples will be required in order to characterize cancer at a molecular level and identify the disease subgroups that should receive different treatments. The use of FFPE tissue is important for enabling such studies. [16] Here we tested AmpliSeq and nCounter custom CNV panels in FFPE gastric cancer samples to determine if they are applicable in archival clinical samples for personalized targeted therapies.

## Materials and Methods

### Samples

Tumor cell percentage with more than 75% were dissected under microscopy from 4 mm unstained sections by comparison with a H&E stained slide, and genomic DNA was extracted using a Qiagen DNA FFPE Tissue Kit (Qiagen, Hilden, Germany) according to the manufacturer’s instructions from 96 patients with advanced gastric cancer. After extraction, we measured concentration as well as 260/280 and 260/230 nm ratio by spectrophotometer (ND1000, Nanodrop Technologies, ThermoFisher Scientific, MA, USA). Each sample was then quantified with the Qubit fluorometer (Life Technologies, Carlsbad, California). Genomic DNA with >10 ng measured by Qubit fluorometer was subjected to library preparation and seven samples failed to construct libraries and were excluded from this study. Finally, 89 cases were finally analyzed and included 31 female and 58 male patients. Table 1 lists the clinical and pathologic features of the patients in this study. Recurrence or metastasis developed in 11 patients with median follow-up period of 76 months (range 5.5–149.3). The study was approved by the institutional review board (IRB) at Samsung Medical Center. All clinical investigation was conducted according to the principles expressed in the Declaration of Helsinki. The written informed consent was waived by the IRB due to retrospective analysis and anonymous data. Samples were collected as part of a routine medical procedure and were collected by the authors for this study. Samples from deceased patients or live patients were all de-identified, including removal of any and all demographic information, prior to analysis and informed consent form was waived by the IRB.

**Table 1:** Clinical and pathological characteristics of 89 patients with gastric cancer.

		Number of cases (n = 89)
Gender	F	31
	M	58
Age	Mean	53
	Median	55
Lauren’s classification	Intestinal	27
	Diffuse	60
	Mixed	2
Location	Upper 1/3	11
	Mid 1/3	32
	Lower 1/3	46
pT stage	T1, 2	23
	T3	51
	T4	15
pN stage	N1	41
	N2	44
	N3	4
AJCC/UICC stage (7^th^ ed)	I	1
	II	22
	III	66
Recurrence and/or distantmetastasis	present	11
	absent	78
Follow up period (months)	Median (range)	76 (5.5–149.3)

### Ion AmpliSeq cancer panel v2

We used the Ion AmpliSeq Cancer Panel v2 (Ion Torrent) to detect frequent somatic mutations that were selected based on literature review. It examines 2855 mutations in 50 commonly mutated oncogenes and tumor suppressor genes (Table S1). First, 10 ng of DNA from each of 89 FFPE tumor samples underwent single-tube, multiplex PCR amplification using the Ion AmpliSeqCancer Primer Pool and the Ion AmpliSeqKit reagents (Life Technologies). Treatment of the resulting amplicons with FuPa Reagent partially digested the primers and phosphorylated the amplicons. The phosphorylated amplicons were ligated to Ion Adapters and purified. For barcoded library preparation, we substituted barcoded adapters from the Ion Xpress Barcode Adapters 1–96 Kit for the non-barcoded adapter mix supplied in the Ion AmpliSeq Library Kit. The ligated DNA underwent nick-translation and amplification to complete the linkage between adapters and amplicons and to generate sufficient material for downstream template preparation. Two rounds of Agencourt AMPure XP Reagent binding at 0.6 and 1.2 bead-to-sample volume ratios removed input DNA and unincorporated primers from the amplicons. The final library molecules were 125∼300 bp in size. We then transferred the libraries to the Ion OneTouch System for automated template preparation. Sequencing was performed on the Ion PGM sequencer according to the manufacturer’s instructions. We used IonTorrent Software for automated data analysis.

To measure the sensitivity and specificity of the Ion AmpliSeq cancer panel, whole exome sequencing results from 4 gastric cancer samples with known mutation status were used [17].

### nCounter Copy Number Variation CodeSets

For detection of CNV, nCounter Copy Number Variation CodeSets were used with 300 ng purified genomic DNA extracted from 2–3 sections of 4-µm-thick FFPE representative tumor blocks using QIAamp DNA FFPE Tissue Kit (Qiagen, Hilden, Germany). DNA was fragmented via AluI digestion and denatured at 95°C. Fragmented DNA was hybridized with the codeset of 86 genes in the nCounter Cancer CN Assay Kit (Nanostring Technologies) for 18 hours at 65°C and processed according to the manufacturer’s instructions. The nCounter Digital Analyzer counted and tabulated the signals of reporter probes and average count numbers of >3 were called and confirmed by IHC, FISH or real-time PCR.

### IHC for HER2, EGFR (HER1) and CCNE1

For validation of CNV results obtained from nCounter, we performed IHC for HER2 in all cases, and EGFR and CCNE1 in selected cases. After deparaffinization and rehydration, 4 mm sections on silane-coated slides were immunostained for HER2. The HercepTest (Dako, Glostrup, Denmark) was used according to the manufacturer’s guidelines as previously described. [18] For EGFR we used anti-NCL-L-EGFR-384 mouse monoclonal primary antibody (1∶100 dilution; Novocastra/Vision Biosystems, Newcastle, UK) and for CCNE1 we used anti-CCNE1/Cyclin E1 Antibody (clone HE12; 1∶200 dilution; Thermo Fisher Scientific, MA). The Ventana BenchMark XT automated slide-processing system was used according to the manufacturer’s protocol. An expert pathologist (KMK) evaluated the results.

### FISH for HER2

FISH was performed using dual-color DNA-specific probes from PathVision™ (Abbott/Vysis: LSI HER2 SpectrumOrange™ and CEP 17 SpectrumGreen™) as previously described in cases with equivocal HER2 overexpression. [19] We counted the hybridization signals in 20 nuclei per sample under a fluorescent microscope (Zeiss Axioskop) using filter sets recommended by Vysis (DAPI/Spectrum Orange dual bandpass, DAPI/Spectrum Green dual bandpass). All overlapping nuclei were excluded, and only nuclei with a distinct nuclear border were evaluated. *HER2* gene was considered amplified when the FISH signal ratio of *HER2/CEP17* was greater than or equal to 2.0 [20].

### Real-time PCR for KRAS and MET amplification

We used DNAs obtained from FFPE gastric carcinoma tumor tissues. The reaction mixture contained 2 uL genomic DNA template, 10 uL of Taqman universal PCR master mixture (Applied Biosystems Inc, Foster City, CA) and 0.2 uM of each primer. For accurate detection of CN alterations, we analyzed three different regions of the *KRAS* gene: a region within intron 1 (TaqMan Copy Number Assay Hs06943812_cn), a region within intron 2 (Hs002534878_cn), and a region within exon 6 (Hs02739788_cn). For *MET* gene, we used the primers as previously described [21].

We measured copy number gain using the following profile: 2 min at 50°C, denaturation at 95°C for 10 min, followed by 40 cycles of 95°C for 15 sec and 60°C for 1 min. We determined relative quantification using the 7900 HT fast real-time PCR system in quadruplicate. An RNaseP assay kit (Applied Biosystems) was used as a control. After amplification, we imported the experiment results containing threshold-cycle values for the copy number and reference assay into the CopyCaller Software (Applied Biosystems) for post-PCR data analysis as previously described. [22] We assigned the CN gain status and the number of *KRAS* copies based on the concordance of the results in at least two of the three probes.

### Analytical methods

**Figure 1:**
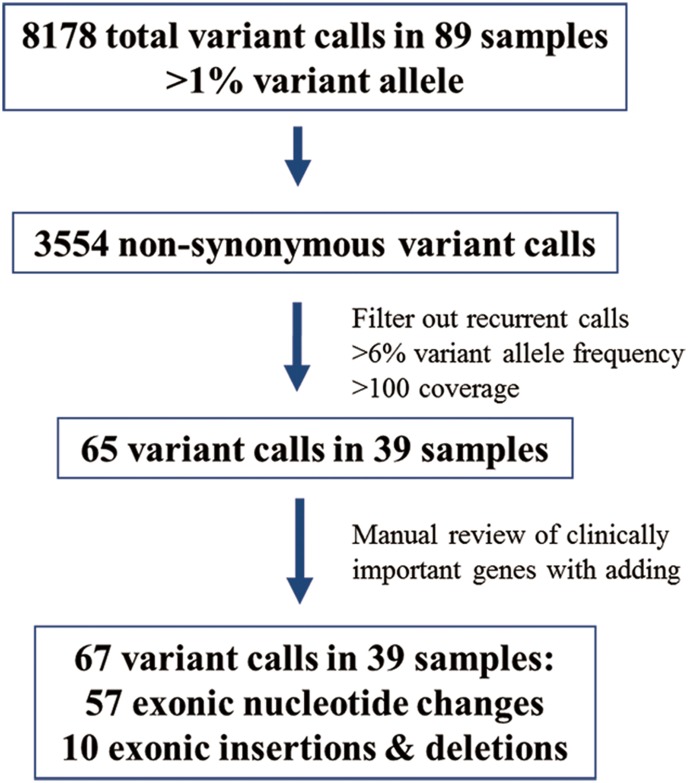
Summary of variant call processing.

We excluded all synonymous changes after an automated mutation-calling algorithm was used to detect supposed mutations. Recurrent calls in more than 10 of 89 samples were regarded as false positive and were excluded. We used cutoff values of more than 6% variant frequency and more than X100 coverage to detect true mutational changes in accordance with previous studies and our own experience. [9], [10] We filtered out single-nucleotide polymorphisms after manual review of each polymorphism in the Catalogue of Somatic Mutations in Cancer (COSMIC, http://cancer.sanger.ac.uk/cancergenome/projects/cosmic) (Figure 1). For well-known genes mutated in gastric carcinomas (*TP53*, *APC*, *PIK3CA*, *STK11*, *CDKN2A*, *KRAS*, *HRAS*, *BRAF* and *CTNNB1*), a manual review of automated calling results was performed to catch deleterious mutations with slightly low-variant frequency.

## Results

### Results of the Ion AmpliSeq cancer panel

The concentrations of DNAs, their concentration fold, average coverage of the samples, total numbers of bases, >Q20 bases, reads, mean read length, mapped reads, on-target rate (%), mean depth and uniformity of the results are described in Table S2. In total we obtained 8178 variant calls from 89 samples, among them 3554 calls were non-synonymous changes. After filtering out recurrent calls, <6% of variant-allele frequency, <100X coverage and those in intron region, 65 variant calls were selected. Additionally, we reviewed the automated calls in well-known mutations such as *BRAF*, *KRAS* and *PIK3CA* and could save two variant calls, which were excluded during the filtering processes. Thirty-nine of the 89 samples (43.8%) harbored at least one mutation (Figure 1). Two cases showed 22 and 5 mutations, respectively. The latter case harbored *MLH1* somatic mutation [missense mutation in exon 20: c.1147A>G(p.M383 V)] and *MLH1* promoter hypermethylation with MLH1 protein losses by IHC using the previously described methods, [23] suggesting hypermutated tumor. However, although the mutations found in the former case passed variant frequency and coverage cut offs, those mutations were not confirmed by Sanger sequencing, suggesting false positive in this case due to poor quality of DNA. So, this case was excluded from final analyses of the results. Frequently detected somatic mutations included *TP53* (24 cases, 27.0%), *APC* (9 cases, 10.1%), *PIK3CA* (5 cases, 5.6%), %), *KRAS* (3 cases, 3.4%), *SMO* (4 cases, 4.5%), *STK11* (3 cases, 3.4%), *CDKN2A* (3 cases, 3.4%), and *SMAD4* (3 cases, 3.4%) as shown in Table 2. Table 2 also summarized the amino acid changes in frequently mutated genes. We identified 19 patients (21.3%) with two or more unique and concomitant somatic mutations.

**Table 2:** Frequency of mutations and amino acid changes in 89 gastric carcinomas.

Gene	N*	%	Amino acid change (N, %)
*TP53*	24	27.0	R248Q (N = 3, 3.3%)^§^
			R248W (N = 1, 1.1%)
			R213fs*34 (N = 1, 1.1%)
			R213* (N = 1, 1.1%)
			R273H (N = 1, 1.1%)
			R273C (N = 1, 1.1%)
			R175H (N = 2, 2.2%)
			R185R (N = 1, 1.1%)
			R342* (N = 1, 1.1%)
			C135C (N = 1, 1.1%)
			C135fs*35 (N = 2, 2.2%)
			C176S (N = 1, 1.1%)
			D208V (N = 1, 1.1%)
			G245R (N = 1, 1.1%)
			T626C (N = 1, 1.1%)
			L206fs*41 (N = 1, 1.1%)
			V173A (N = 1, 1.1%)
			Y236C (N = 1, 1.1%)
*APC*	9	10.1	K1359E (N = 1, 1.1%)^§^
			K1363E (N = 1, 1.1%)
			P1433L (N = 1, 1.1%)
*PIK3CA*	5	5.6	E545K (N = 2, 2.2%)
			N1044K (N = 1, 1.1%)
			E1037K (N = 1, 1.1%)
			H1047R (N = 1, 1.1%)
*KRAS*	3	3.4	G13V (N = 2, 2.2%)
			G12V (N = 1, 1.1%)
*SMO*	3	3.4	E518K (N = 1, 1.1%)
			E208K (N = 1, 1.1%)
			R512H (N = 1, 1.1%)
*STK11*	3	3.4	S31F (N = 1, 1.1%)^§^
			T32I (N = 1, 1.1%)
*CDKN2A*	3	3.4	T79I (N = 1, 1.1%)
			H66R (N = 1, 1.1%)
			C315A (N = 1, 1.1%)
*SMAD4*	3	3.4	R361H (N = 1, 1.1%)
			M447I (N = 1, 1.1%)
			Q448X (N = 1, 1.1%)
*CDH1*	2	2.2	L343P (N = 1, 1.1%)
			G382D (N = 1, 1.1%)
*FBXW7*	2	2.2	R393* (N = 1, 1.1%)^§^
*ATM*	1	1.1	Y861H (N = 1, 1.1%)
*CTNNB1*	1	1.1	T41A (N = 1, 1.1%)
*ERBB2*	1	1.1	V842I (N = 1, 1.1%)
*FGFR2*	1	1.1	G906A (N = 1, 1.1%)
*FGFR3*	1	1.1	A369A (N = 1, 1.1%)
*KDR*	1	1.1	W1143X (N = 1, 1.1%)
*MLH1*	1	1.1	A424G (N = 1, 1.1%)
*PTEN*	1	1.1	R15S (N = 1, 1.1%)
*SMARCB1*	1	1.1	H177Y (N = 1, 1.1%)
*RET*	1	1.1	A641T (N = 1, 1.1%)

*N, total number of samples with mutation, ^§^INS/DEL in the remaining cases.

In four gastric cancer samples with known mutation frequencies determined by whole exome sequencing and confirmed by Sanger sequencing, we identified somatic mutations in *TP53*, *ERBB4* and *CTNNB1* with no false-positive calls in other genes (Table S3).

### Amplification by nCounter and validation by IHC, FISH or real-time PCR

**Figure 2:**
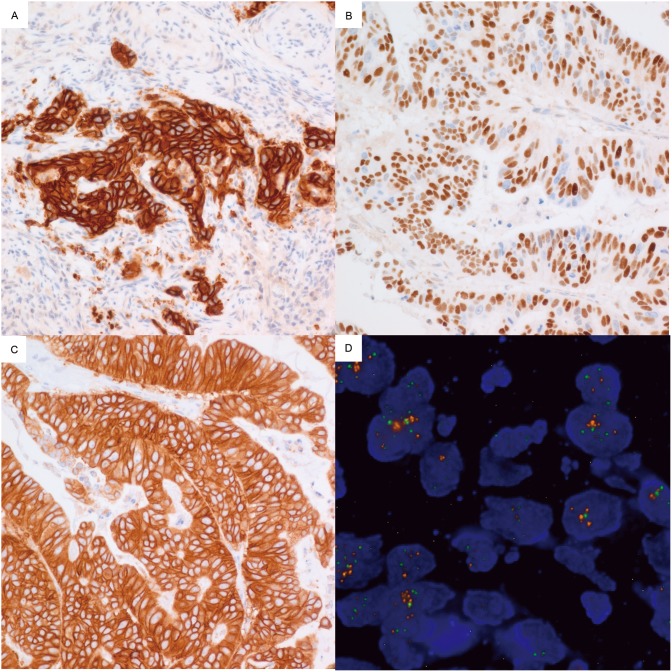
Immunohistochemical staining for EGFR, CCNE1 and HER2, and FISH for HER2. Cases with copy number increases showed strong positive for EGFR (A), CCNE1 (B) and HER2 (C). A case with HER2 2+ by immunohistochemistry reveals amplification of HER2 genes in FISH (D).

Amplifications of *HER2*, *CCNE1*, *MYC*, *KRAS* and *EGFR* genes were observed in 8 (8.9%), 4 (4.5%), 2 (2.2%), 1 (1.1%) and 1 (1.1%) cases, respectively (Table 3). We did not observe amplification of *MET*, *FGFR2*, *CDK4* and CDK6 in any of the cases. In cases with amplification, IHC for HER2, EGFR and CCNE1 showed overexpression of proteins in the tumor cells (Figure 2A, B and C). In one case with HER2 2+ by HercepTest, FISH showed heterogeneous amplification of *HER2* genes (Figure 2D).

**Table 3:** Gastric cancers with copy number variation (CNV) detected by nCounter.

Gene	Number of samples with CNV	Range of CNV (mean)
	(% in total 89 samples)	
*ERBB2* *	8 (8.9%)	9–62 (31.9)
*CCNE1*	4 (4.5%)	8–22 (12.8)
*MYC*	2 (2.2%)	13–38 (25.5)
*EGFR*	1 (1.1%)	7
*KRAS*	1 (1.1%)	18

*All samples have proven to show positivity in immunohistochemical staining (8 samples: 3+; 1 sample: 2+; 1 sample: 1+).

In real-time PCR for *KRAS*, one case with amplification showed increased copy numbers (36, 37 and 49); in cases that were negative for *KRAS* amplification, there was no increase of copy numbers (0.9 to 2.4, mean 1.4).

For *MET* gene, we find no positive case because of their rarity [21]. Therefore, we used additional ten (five each amplified and non-amplified) gastric cancer samples and *MET* amplified gastric cancer cell lines (MKN45 and SNU5) with known copy numbers and mRNA amounts. CNVs detected by nCounter correlated well with copy numbers detected by real-time PCR (Table S4) and mRNA levels of *MET* gene (Pearson’s correlation test; r = 0.874, p = 0.001) (Figure S2).

## Discussion

By using Ion AmpliSeq v2 we found that 39 out of 89 advanced gastric adenocarcinoma samples contained somatic mutations, demonstrating that this platform is easily applicable in archival FFPE tissue samples. *TP53* was the most frequently found mutation, followed by *APC*, *PIK3CA* and *KRAS*. Moreover, our custom CNV panel successfully detected CN increases of *HER2*, *CCNE1*, *MYC*, *EGFR* and *KRAS* genes, which we confirmed by IHC and real-time PCR.

Mutational frequencies in the COSMIC database reveal substantial similarities to the data we obtained from this study: *TP53* (32%), *PIK3CA* (10%), *KRAS* (6%), *APC* (6%), *CTNNB1* (5%), *CDKN2A* (5%), *FBXW7* (5%), *SMO* (4%), *ERBB2* (2%) and *STK11* (2%). Recent whole exome sequencing studies on gastric adenocarcinoma showed somewhat higher frequencies of *TP53* (36% and 73%) and *PIK3CA* (14% and 20%) mutations compared to our results. [24], [25] Although our mutation frequencies were lower when compared to exome sequencing results, there was a significant increase when compared to our previous data on mass spectrometry-based OncoMap v4. [26] Both AmpliSeq and OncoMap detect mutations in hotspot regions, which explain findings of less frequent mutation in some oncogenes and tumor suppressor genes. Figure S1 compares AmpliSeq v2 and OncoMap v4 in detectable mutational profiles. Previous OncoMap tests in 237 gastric adenocarcinomas revealed that *PIK3CA* mutations were frequent in advanced stages of disease (5.1% in Stage IV; 6.4% in stage II/III; 2.4% in stage IB). [26] In this study we observed three *PIK3CA* mutations in patients with stage III and two in stage II disease, supporting their biological role in tumor progression. We also observed *HER2* (*ERBB2*) c.2524G>A (V842I) mutation in a case of gastric cancer. In preclinical studies, cell lines harboring the V842I mutation were resistant to trastuzumab, but were sensitive to irreversible HER2 inhibitor, neratinib [27].

Semiconductor-based sequencing has fundamental differences in sensing and signal transduction compared to mass spectrometry-based sequencing. Instead of using optical methods to detect nucleotide changes, the semiconductor-based technique senses pH changes by release of protons (H^+^) when nucleotides integrate into the growing DNA strand. [8] Therefore, there is a significant reduction in the cost and the time required for data processing compared to other NGS platforms. Providing fast and accurate information on mutations at low cost is crucial for patients with highly aggressive cancers, including gastric cancer.

In this study, we manually reviewed the automated calls in well-known mutations after applying cutoff values of frequency and coverage, subsequently adding two calls with low-variant coverage. Although their coverage values did not reach our initial criteria, their frequencies exceeded our first setting (6%) and the quality of the data was good. This emphasizes the importance of manual review after automated screening. Recently published results using AmpliSeq as the analyzing platform also emphasize compensation of screening data with manual review [9], [10].

Personalized targeted therapy for advanced cancers primarily relies on the concept of “oncogene addiction,” in which multiple genetic abnormalities are addicted to one or a few genes for tumor cell maintenance and survival. [28] An open-label, international, phase 3, randomized controlled ToGA (Trastuzumab for Gastric Cancer) trial indicated that trastuzumab in combination with chemotherapy is a new standard option for patients with HER2-positive advanced gastric or gastro-esophageal junction cancer. [29] A preclinical trial showed that a subset of gastric cancers with *EGFR* or *MET* amplification and overexpression respond to cetuximab or MET receptor tyrosine kinase inhibitor therapy. [30] Additionally, amplifications of cell cycle mediator *CCNE1* suggest the potential for therapeutic inhibition of cyclin-dependent kinases in gastric cancers. [31] Screening amplified genes for targeted therapy with high-throughput technology is very important. Traditional methods such as FISH and array comparative genomic hybridization suffer from low resolution of genomic regions, high cost and are labor- and time-consuming. [32] In this first study on nCounter CNV analyses, we found that this technology is applicable in FFPE clinical samples and we validated the results by IHC, FISH and real-time PCR. Although we did not validate all the genes used in the custom primers, validation results in several selected genes were remarkable.

In summary, we successfully performed semiconductor-based sequencing and nCounter CNV analyses in FFPE tissue specimens from 89 gastric adenocarcinomas. High-throughput sequencing and CNV screening in archival clinical samples enables faster, more accurate and cost-effective detection of hotspot mutations and CNV in genes. In the era of personalized genomic medicine, we plan to use these tools to screen for gastric cancer patients who may benefit from targeted therapies.

## Supporting Information

Figure S1 **Comparison of coverage of Ion AmpliSeq v2 cancer panel versus Oncomap v4.**
(TIF)

Figure S2 **Plots of correlation between** ***MET*** **CNVs detected by nCounter and mRNA levels of** ***MET*** **gene by real-time PCR.**
(TIF)

Table S1 **The Gene List for the Ion Torrent AmpliSeq Cancer Panel.**
(XLS)

Table S2 **The concentrations of DNAs, their concentration fold, average coverage of the samples, total numbers of bases, >Q20 bases, reads, mean read length, mapped reads, on-target rate (%), mean depth and uniformity.**
(XLS)

Table S3 **Somatic mutations in** ***TP53*** **,** ***ERBB4*** **and** ***CTNNB1*** **.**
(XLS)

Table S4 **CNVs detected by nCounter correlated well with copy numbers detected by real-time PCR.**
(XLSX)
