# Supplementary figures and images for: High-Throughput Sequencing and Copy Number Variation Detection Using Formalin Fixed Embedded Tissue in Metastatic Gastric Cancer
Source: PLoS One. 2014 Nov 5;9(11):e111693. doi: 10.1371/journal.pone.0111693 (PMC4221102; doi:10.1371/journal.pone.0111693)

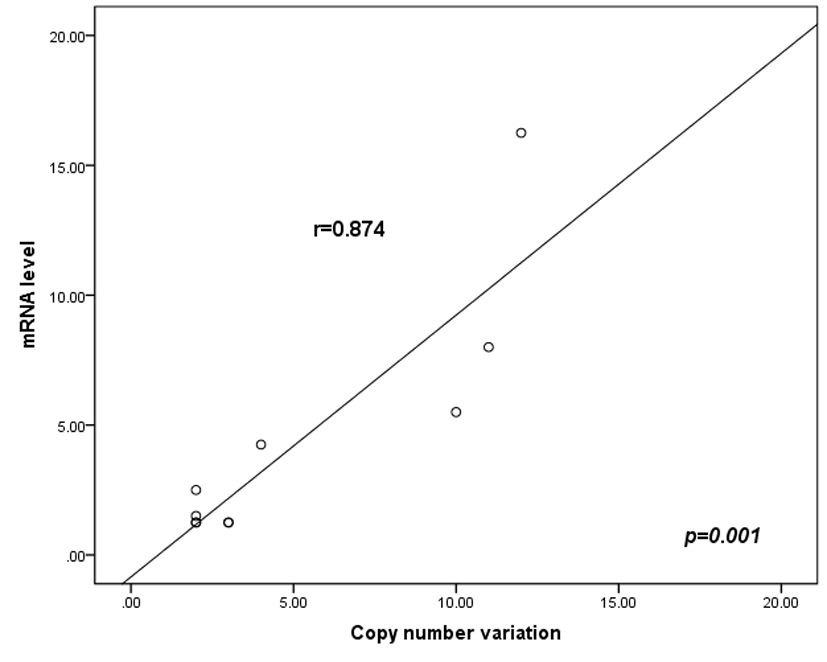

Supplement: Figure S2 — Plots of correlation between MET CNVs detected by nCounter and mRNA levels of MET gene by real-time PCR. (TIF) [file pone.0111693.s002.tif]
